# Impact of Three to Five Rounds of Mass Drug Administration on Schistosomiasis and Soil-Transmitted Helminths in School-Aged Children in North-Central Nigeria

**DOI:** 10.4269/ajtmh.21-1207

**Published:** 2022-05-16

**Authors:** Emily Griswold, Abel Eigege, Solomon Adelamo, Bulus Mancha, Nwodu Kenrick, Yohana Sambo, Joseph Ajiji, Gideon Zam, Jacob Solomon, Rita O. Urude, Jonathan Kadimbo, Jacob Danboyi, Emmanuel Miri, Andrew W. Nute, Lindsay Rakers, Obiageli Nebe, Chukwuma Anyaike, Paul Weiss, Gregory S. Noland, Frank Richards

**Affiliations:** ^1^ The Carter Center, Atlanta, Georgia;; ^2^ The Carter Center, Jos, Nigeria;; ^3^ Plateau State Ministry of Health, Jos, Nigeria;; ^4^ Nasarawa State Ministry of Health, Lafia, Nigeria;; ^5^ Federal Ministry of Health, Abuja, Nigeria;; ^6^ Emory University, Atlanta, Georgia

## Abstract

Nasarawa and Plateau states of north-central Nigeria have implemented programs to control schistosomiasis (SCH) and soil-transmitted helminths (STH) in children since the 1990s. Statewide mapping surveys were conducted in 2013, when 11,332 school-aged children were sampled from 226 schools. The local government areas (LGAs) then received varying combinations of mass drug administration (MDA) for the next 5 years. We revisited 196 (87%) schools in 2018 plus an additional six (202 schools in total), sampling 9,660 children. We calculated overall prevalence and intensity of infection and evaluated associations with gender; age; behaviors; water, sanitation, and hygiene (WASH); and treatment regimen. Urine heme detection dipsticks were used for *Schistosoma hematobium* in both surveys, with egg counts added in 2018. Stool samples were examined by Kato-Katz for *Ascaris lumbricoides*, *Trichuris trichiura*,* Schistosoma mansoni*, and hookworm. Schistosomiasis prevalence among sampled students dropped from 12.9% (95% confidence interval [CI]: 11.1–14.9%) to 9.0% (95% CI: 7.5–10.9%), a statistically significant change (*P* < 0.05). In 2018, eight LGAs still had > 1% of children with heavy-intensity schistosome infections. Prevalence of STH infection did not significantly change, with 10.8% (95% CI: 9.36–12.5%) of children positive in 2013 and 9.4% (95% CI: 8.0–10.9%) in 2018 (*P* = 0.182). Heavy-intensity STH infections were found in < 1% of children with hookworm, and none in children with *A. lumbricoides* or *T. trichiura* in either study. The WASH data were collected in 2018, indicating 43.6% of schools had a latrine and 14.4% had handwashing facilities. Although progress is evident, SCH remains a public health problem in Nasarawa and Plateau states.

## INTRODUCTION

An estimated 1.5 billion people around the world have infections with soil-transmitted helminths (STHs) specifically *Ascaris lumbricoides*, *Trichuris trichiura*, or either hookworm: *Necator americanus* and *Ancylostoma duodenale*.^1^ Schistosomiasis (SCH), caused by *Schistosoma mansoni* or *Schistosoma hematobium*, has been estimated to affect more than 200 million people.^2^^,^^3^ School-aged children (ages 5–14 years) often carry the most burden of these infections, which contribute to malnourishment, anemia, and other morbidities.^4^ Nigeria is home to the largest number of people in the world in need of treatment of SCH (> 25 million),^5^ and the fourth-largest number of children in need of treatment of STHs (> 48 million).^6^

Nasarawa and Plateau states are located in north-central Nigeria and consist of 30 local government areas (LGAs, or administrative districts) across both states, 13 in Nasarawa and 17 in Plateau. Their combined population in 2018 was around 7 million. The SCH control program in these states began in 1999, focusing on urinary SCH. Initial mapping in 1999 in two LGAs found dipstick positivity (a marker for *S. hematobium* infections) in children ranged from 0% to 87%.^7^ The situation was similar 5 years later, when studies in 13 LGAs found village dipstick positivity ranging from 0% to 100%, with one LGA above 50% overall prevalence.^8^ These results indicated praziquantel mass drug administration (MDA) was needed.^9^ However, limited funds and medicines led to relatively small proportions of the population receiving treatment, reaching no more than 200,000 children per year from 1999 through 2007, a total of 1.15 million doses, either via stand-alone treatment or integrated into the MDAs of onchocerciasis and/or lymphatic filariasis (LF) programs.^10^ These treatments were effective at reducing hematuria.^7^ This changed in 2008 with a major drug donation by E-Merck through the WHO, allowing treatment to expand in frequency, reach, and to target intestinal as well as urinary SCH.^11^ From 2008 onward, more than 1.1 million treatments were administered each year.

Soil-transmitted helminths were addressed via the LF program’s use of albendazole and ivermectin in community-wide MDA, which took place throughout the two states. Although some LGAs began MDA in 2000 and some ended in 2009, most were treated from 2003 to 2012.^12^^,^^13^ At that time, both states had reached the point where they could stop MDA for LF and transitioned the STH program to focus on school-aged children using the drug mebendazole. Out of the 30 LGAs, 12 were endemic for onchocerciasis and continued annual community-wide MDA with ivermectin through 2017.^14^ As the LF program ended completely in 2013, it was important to establish future treatment needs; intestinal and urinary SCH were assessed at the same time in widespread school-based surveys.

Based on the prevalence results in the 2013 survey, LGAs (the administrative units for treatment decisions) were stratified to receive MDA either annually or every other year for SCH and STHs between 2013 and 2018. All 30 LGAs received mebendazole for STH in 2014, 2016, and 2018, whereas 11 (37%) received it every year. All 30 LGA received praziquantel in 2013, 2014, 2016, and 2018; 6 (20%) received it every year. Only one LGA, Karu in Nasarawa state, received both drugs each year. More detailed treatment histories are available in Supplemental Figure 1. Administratively reported coverage was generally above 90% among school-age children but dropped as low as 61% for STH in one round because of shortage of mebendazole tablets. Net school enrollment rates at the state level were between 53.2% and 62.4% in the study years,^15^^,^^16^ but this information was not available for the sampled schools and surrounding communities.

The 2018 follow-up survey was conducted in the same schools to measure any changes in disease burden and to determine whether the MDA frequency in the various LGAs should change. This study presents the results of the 2013 surveys along with the 2018 surveys and offers conclusions regarding the impact of MDA during those years.

## MATERIALS AND METHODS

### Ethics.

Ethical clearance was obtained from the Federal Ministry of Health, Nigeria, after submitting the survey proposal, which contained the study plan, consent/assent forms, and permission forms from parents/guardians and the head teacher. On arrival in the school, the consent of the school was sought after which the assents of the selected pupils were recorded.

### Study design.

The design of this study was to compare samples taken from schoolchildren in 2013 and 2018 to determine changes in prevalence and intensity of *A. lumbricoides*, *T. trichiura*, hookworm and *S. mansoni*, and in prevalence of *S. hematobium.* The 2013 survey was conducted in April–June, whereas that of 2018 was June–July. The 2013 MDA was July–November, whereas that of 2018 was August–November.

In 2013, the state Ministries of Health and Education provided lists of schools in each LGA. From this list, seven to nine schools were randomly selected from each of the 30 LGAs for a total of 220 schools. Six schools were then added because they had shown high rates of positivity during heme dipstick testing between 1999 and 2007 (unpublished data). During analysis, these “high-risk” schools were placed in their own stratum as they were not randomly selected to represent the schools from the original sampling frame. During the follow-up study in 2018, security issues prevented sampling at 20 schools; we therefore surveyed 196 of the 226 schools from the 2013 sample, randomly selecting six additional schools to balance the sample. The 2018 sample included the same six high-risk schools as in 2013. Approximately, 50 children aged 6–14 years were selected per school in each time point.

A sample size of 50 individuals of both sexes (about 50% male and 50% female) was intended. The students aged 6–14 years in the selected schools were assembled in two lines, one for boys and one for girls. Children were selected systematically from within these two lines, using a sampling interval derived from the target sample size and the number of children present.

### Sample collection and analysis.

Each selected pupil was given two specimen bottles, one each for stool and urine. The stool samples were processed using the Kato-Katz technique. Briefly, samples were processed in the mobile laboratory by collecting approximately 40–45 mg of stool in a plastic template using a spatula over a nylon mesh. Hydrophilic cellophane soaked in glycerol malachite green solution was used as a cover slip on the stool sample placed on a glass slide. Duplicate slides were done for stool samples and read by the same microscopist for egg counts, with the average eggs per slide taken to calculate the eggs per gram (epg) of stool for each organism. Infection was defined as the presence of at least one egg of *A. lumbricoides*, *T. trichiura*, *S. mansoni*, *N. americanus*, or *A. duodenale*.

Urine samples were first tested with a dipstick (Combi-9), which was read and graded within 60 seconds according to the manufacturer’s guideline. Ten percent of dipstick-positive urine samples were immediately preserved in formalin for later microscopy. Urine filtration was used for egg quantification and examination by wet mount. Ten milliliters of urine were drawn from the specimen bottle into a 10-mL syringe. The gasket with a membrane filter was placed securely at the tip of the syringe. Urine was then flushed through the gasket. The membrane was retrieved with forceps and placed on a clean slide, to which one to two drops of lugols iodine solution was added, then covered with a slip. This was viewed under the microscope to count any eggs of *S. hematobium*.

We categorized infections according to the WHO guidance.^17^ Thresholds for heavy-intensity infections were ≥ 400 epg of stool for *S. mansoni*, ≥ 50,000 for *A. lumbricoides*, ≥ 4,000 for hookworm, and ≥ 10,000 for *T. trichuria*. As urine filtration and egg counting were not done in 2013, we can only assess intensity of infection of *S. hematobium* in 2018; heavy infections were set at ≥ 50 eggs per 10 mL of urine.

Dipstick tests were categorized as negative, +, ++, and +++ in 2013. “Trace” was added between negative and + in 2018. Schistosomiasis cases were defined as any of the following: dipstick result of “Trace” or higher, any *S. hematobium* eggs found in the urine sample, or any *S. mansoni* eggs in the stool sample. Schistosome infections were defined in 2013 as having the presence of hematuria or *S. mansonie* eggs in the stool; this definition expanded in 2018 to include the presence of *S. hematobium* eggs in urine. A child with a positive result by any of these tests was said to be SCH positive. Soil-transmitted helminth infection was defined as the presence of *A. lumbricoides*, *T. trichiura*, or hookworm in the stool. A child with one or more of these organisms in their stool was said to be STH positive.

### Data collection and analysis.

Data were collected on Android tablets using Open Data Kit-based applications and cloud-based databases, namely, the LINKS system in 2013 and NEMO (https://getnemo.org/) in 2018.^18^ Standardized forms were used to gather information about the school and the individual student. Data on water, sanitation, and hygiene (WASH) facilities were collected in this study for 198 of 202 schools in 2018.

School- and student-level questionnaires were administered in 2018. Data collectors observed the water and sanitation infrastructure at the school and reviewed its treatment records. Students were asked about their behaviors regarding water, urination, and defecation. Interviewers observed whether they were wearing shoes. In addition, the survey team inspected and graded water and sanitation facilities at the surveyed schools using questionnaires provided by the Federal Ministry of Health.

Data from both surveys were consolidated in Microsoft Excel (Microsoft Corp, Redmond, WA) and then analyzed in STATA v. 15.1 (StataCorp LLC, College Station, TX). We used procedures for complex survey designs where indicated, using the school as the primary sampling unit, stratified by LGA with self-representing high-risk schools placed into their own strata. We examined the data for possible associations between SCH or STH and the presence of WASH facilities at schools, controlling for treatment regimen, age, gender, and high-risk behaviors reported by students (e.g., activities involving water for SCH, and not wearing shoes for STH). Single- and multivariate models were created with purposively chosen variables, testing for associations with logistic regression. Comparisons between proportions were made with χ^2^ tests, using postestimation procedures to account for the survey design.

## RESULTS

### Study population.

**Figure 1.**
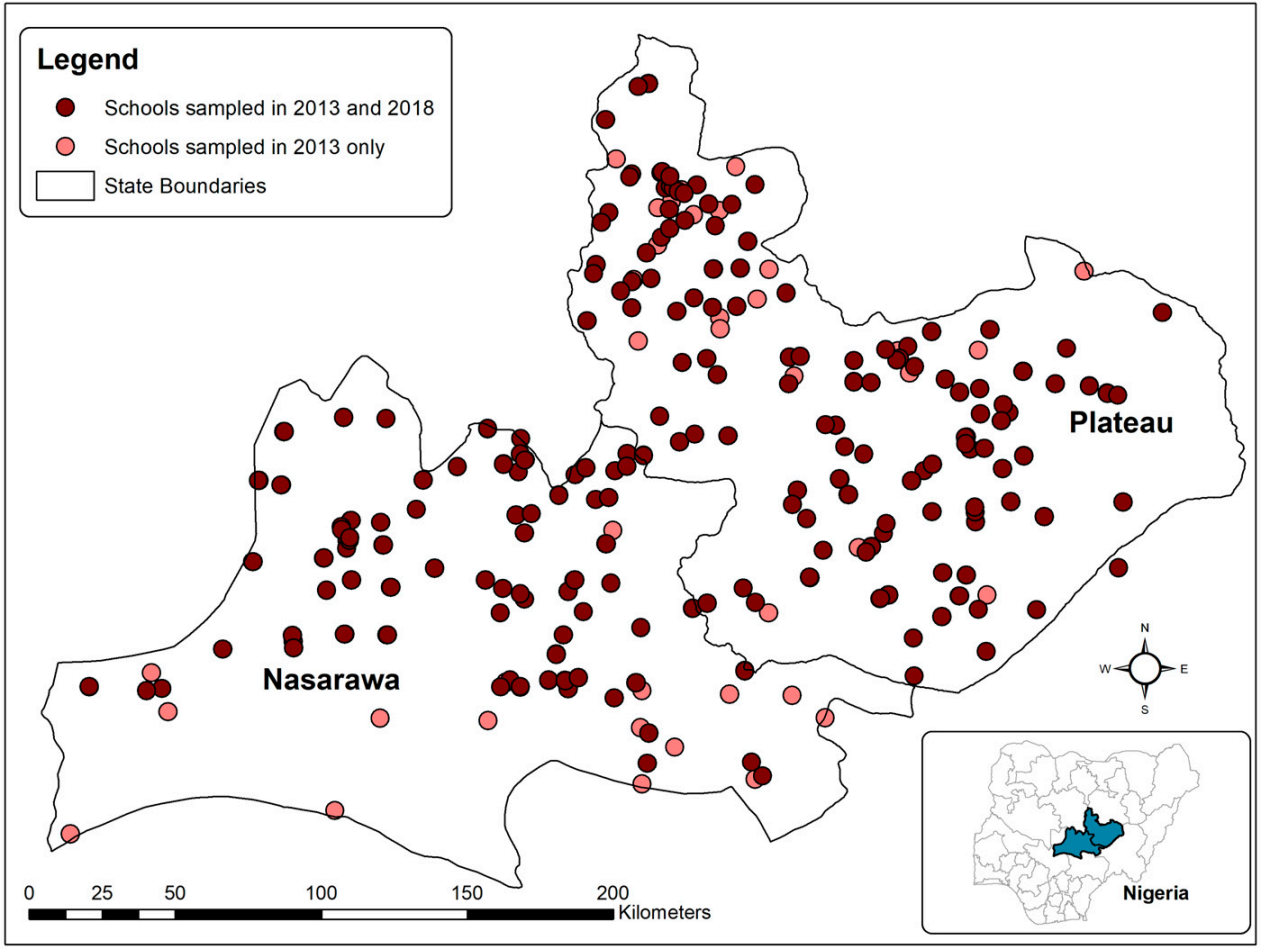
Locations of sampled schools in Nasarawa and Plateau states, Nigeria. This figure appears in color at www.ajtmh.org.

The locations of schools sampled in both years are shown in Figure 1. A total of 20,992 children were sampled across the two studies, 11,332 aged 6–15 years in 2013 (15-year-olds were sampled if they were in the same class as 14-year-olds) and 9,660 aged 6–14 years in 2018. There were more boys in both the samples (52.3% in 2013 and 51.2% in 2018), and the mean age was slightly younger in 2018 than in 2013 (Table 1). Of note, 425 (3.6%) children in 2013 and 14 (0.1%) in 2018 declined a stool test. All children received a dipstick test in 2013, and all but 6 of the 9,660 sampled children were tested by dipstick and urine filtration in 2018 (Table 2).

**Table 1:** Participant demographics in 2013 and 2018 evaluations in two central Nigerian states

	Nasarawa		Plateau		Overall	
Demographic	2013	2018	2013	2018	2013	2018
Schools visited	96	79	130	123	226	202
Children sampled	4,872	3,768	6,460	5,892	11,332	9,660
Proportion female	45.7%	48.4%	49.3%	48.2%	47.7%	48.3%
Mean years of age	11.4	10.3	11.3	10.4	11.4	10.4
Primary school attendance	71.0%	70.2%	63.1%	72.6%	n/a	n/a
Secondary school attendance	53.7%	62.4%	53.2%	51.8%	n/a	n/a

The participant age range was 6–15 years in 2013 and 6–14 years in 2018. School attendance is drawn from net attendance ratios in the corresponding Demographic and Health Surveys (DHS).

**Table 2:** Test results for *Schistosoma hematobium* by diagnostic method

Dipstick result	No egg filtration done (2013 only)	Negative by egg count (2018 only)	1–49 eggs/10 mL urine (2018 only)	≥ 50 eggs/10 mL urine (2018 only)	Missing egg count (2018 only)	Total
Negative	9,969	9,174	15	0	–	**19,158**
Trace	–	88	31	4	–	**123**
+	340	61	52	12	–	**465**
++	348	52	59	15	–	**474**
+++	262	40	31	20	–	**353**
Missing	413	0	0	0	6	**419**
**Total**	**11,332**	**9,415**	**118**	**51**	**6**	**20,992**

### Prevalence of infection.

**Figure 2.**
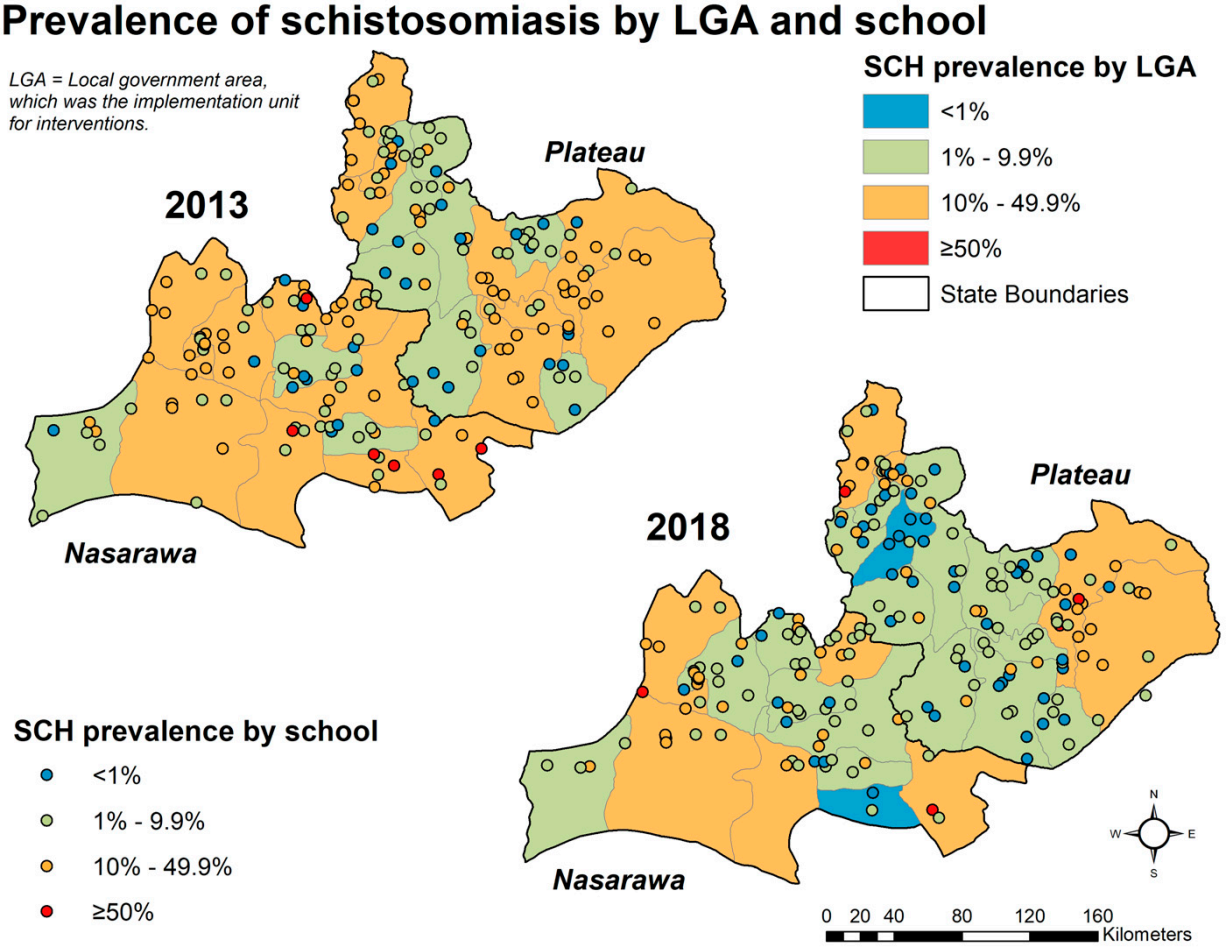
Prevalence of schistosomiasis by LGA and school. This figure appears in color at www.ajtmh.org.

Schistosomiasis—*S. hematobium* or *S. mansoni*—prevalence among all sampled students dropped from 12.9% (95% confidence interval [CI]: 11.1–14.9%, design effect 3.0) in 2013 to 9.0% (95% CI: 7.5–10.9%, design effect 3.0) in 2018, a statistically significant change (*P* < 0.05) (Table 3). Nasarawa state dropped over 4% points, from 15.0% (95% CI: 11.9–18.6%) to 10.6% (95% CI: 8.1–13.8%), whereas Plateau state’s change was more modest, from 11.3% (95% CI: 9.4–13.6%) to 8.0% (95% CI: 6.1–10.4%). However, Plateau’s change in prevalence was statistically significant (*P* = 0.04). Both increases and decreases in SCH prevalence at the LGA and school levels were observed (Figure 2).

**Table 3:** Prevalence of schistosomiasis and STH infections and distribution of causative by organisms in Nigerian children

Organism	2013 Prevalence estimate (95% CI)	2018 Prevalence estimate (95% CI)
*Among all children*		
Schistosomiasis	12.9% (11.1–14.9%)	9.0% (7.4–10.9%)
Only *Schistosoma hematobium*	8.4% (6.8–10.2%)	4.7% (3.7–6.0%)
Only *Schistosoma mansoni*	4.7% (3.7–5.9%)	4.1% (2.9–5.6%)
Both schistosomiasis species	0.3% (0.2–0.6%)	0.2% (0.1–0.4%)
Soil-transmitted helminths		
Only *Ascaris lumbricoides*	1.3% (1.0–1.6%)	0.9% (0.7–1.3%)
Only hookworm	10.0% (8.5–11.8%)	8.5% (7.1–10.2%)
Only *Trichuris trichiura*	0.1% (0.0–0.2%)	0.1% (0.0–0.2%)
More than one organism	0.2% (0.1–0.3%)	0.2% (0.1–0.4%)
*Causative organisms*		
Schistosomiasis infections	*N* = 1,461	*N* = 873
Only *S. hematobium*	62.6% (54.9–69.8%)	52.3% (42.3–62.2%)
Only *S. mansoni*	35.0% (28.0–42.6%)	45.0% (35.2–55.3%)
Both schistosomiasis species	2.4% (1.4–4.2%)	2.6% (1.6–4.2%)
STH infections	*N* = 1,225	*N* = 904
Only *A. lumbricoides*	11.7% (9.3–14.6%)	10.5% (7.6–14.3%)
Only Hookworm	89.2% (86.2–91.6%)	91.0% (87.8–93.5%)
Only *T. trichiura*	0.8% (0.4–1.5%)	1.3% (0.7–2.6%)
More than one organism	1.7% (1.1–2.7%)	2.5% (1.6–4.1%)

CI = confidence interval.

**Figure 3.**
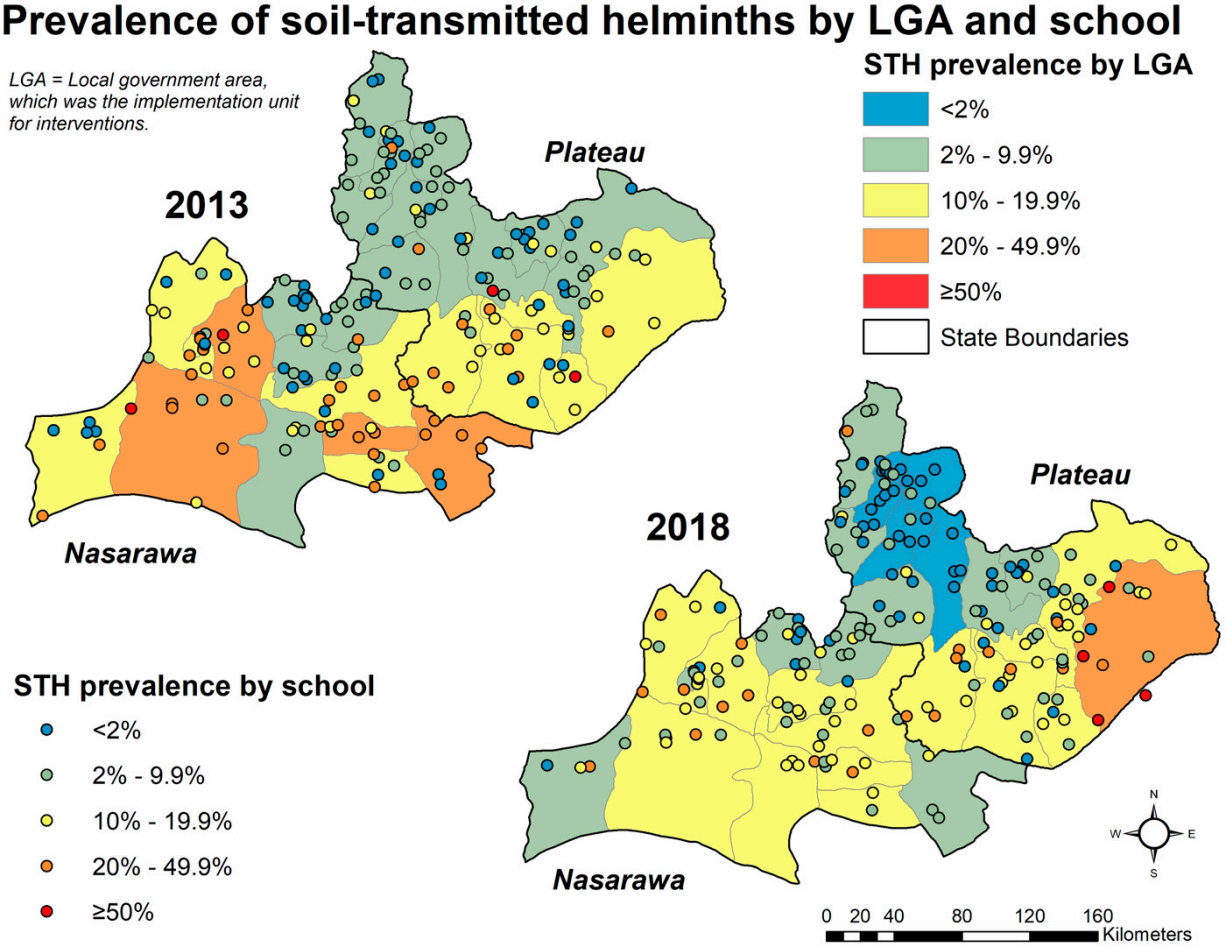
Prevalence of soil-transmitted helminths by LGA and school. This figure appears in color at www.ajtmh.org.

Prevalence of STH infection (any of the three main organisms) did not show statistically significant reductions, with 10.8% (95% CI: 9.36–12.5%, design effect 2.9) of children positive in 2013 and 9.4% (95% CI: 8.0–10.9%, design effect 2.6) positive in 2018 (*P* = 0.182). By state, Nasarawa saw a slight drop from 14.5% (95% CI: 11.7–17.9%) to 11.2% (95% CI: 9.1–13.6%), whereas Plateau state remained steady from 8.0% (95% CI: 6.5–9.8%) in 2013 to 8.2% (95% CI: 6.3–10.5%) in 2018. Neither of these changes were statistically significant (*P* > 0.05). A mix of increases and decreases in STH prevalence were seen (Figure 3).

In 2013, the “high-risk schools” had significantly higher rates of any schistosome infection than the randomly selected schools, 31.3% (95% CI: 17.7–52.9%) versus 12.3% (95% CI: 10.6–14.2%, *P* = 0.01). In 2018, these high-risk schools saw a 21%-point reduction in SCH infection from 31.3% to 10.2% (*P* = 0.03), and the difference with randomly selected schools disappeared; 10.2% (95% CI: 4.4–21.9%) in the high-risk group versus 9.0% (95% CI: 7.4–10.9%, *P* = 0.79). *Schistosoma hematobium* accounted for 78.1% and 87.1% of infections detected in high-risk schools at each timepoint.

Two LGAs saw statistically significant decreases in any schistosome infection. Keana LGA in Nasarawa and Barkin Ladi in Plateau experienced drops from 32.6% to 0.9%, and 7.4% to 0.4%, respectively. For STH, only one LGA, Langtang North in Plateau state, saw a statistically significant increase in its overall STH prevalence, increasing from 4.7% (95% CI: 2.3–9.2%) in 2013 to 15.9% (95% CI: 11.7–21.1%, *P* = 0.00) in 2018. This was driven by an increase in hookworm infection in the LGA from 3.9% (95% CI: 2.2–7.0%) to 14.8% (95% CI: 10.0–2.1%). This change occurred predominantly in boys, who saw 15% point increase, as opposed to only 7% for girls. One LGA, Mangu in Plateau state, saw a significant drop in STH infections from 3.9% (95% CI: 1.6–6.2%) to 0.4% (upper confidence limit [UCL] = 1.2%).

The predominant SCH infection in positive children was *S. hematobium* (65.0% in 2013 to 55.0% in 2018), whereas *S. mansoni* was found in 37.8% versus 47.6% of the samples, respectively. These findings would have been substantially different, had we only used dipsticks or egg counts in 2018 to find *S. hematobium* rather than both (Table 2). Hookworm remained the most common STH among infected children in both surveys (89.2% in 2013 and 91.0% in 2018), followed by ascaris (11.7% and 10.5%), whereas *Trichuris* was the least common (0.8% and 1.3%). Multiple infections were uncommon; only three children were positive for all three STHs, all in 2018. A small proportion of children had both SCH and a STH infection, 2.1% in 2013 and 1.3% in 2018. More detailed results for each organism, both individually and combined, are shown in Table 3.

Schistosomiasis was more common in boys than in girls in both surveys. Overall, 12.2% of boys were positive, compared with 9.9% of girls (*P* < 0.05). This correlation held in both 2013 (14.0% versus 11.6%, *P* < 0.05) and 2018 (10.1% versus 7.9%, *P* < 0.05). Similar associations were found for STH infections; 11.6% of boys and 8.6% of girls were positive for one or more STH (*P* < 0.05). This was true in both 2013 (12.5% versus 8.9%, *P* < 0.05) and 2018 (10.4% versus 8.2%, *P* < 0.05). By organism, the statistically significant associations with gender were for *S. mansoni* only in 2018 (4.8% for boys, 3.8% for girls, *P* = 0.03); *S. hematobium* and hookworm also showed gender-specific associations (*P* < 0.05) but at both timepoints. There were no statistically significant gender-specific correlations with *A. lumbricoides* or *T. Trichuris*.

Children aged 6–10 years were less frequently infected with SCH than their older peers in both 2013 (11.9% versus 13.5%, *P* < 0.05) and 2018 (7.2% versus 11.0%, *P* < 0.05). As with gender, associations with age group for *S. mansoni* were not statistically significant in 2013 (*P* = 0.61) but were in 2018 (*P* < 0.05). Although there was a statistically significant association between the older age group and *S. hematobium* in the combined analysis, with prevalence of 6.8% in older children and 5.2% in younger (*P* < 0.05), this relationship was not significant when either survey year was analyzed individually. There were no statistically significant associations between age group and STH infection.

Ivermectin treatment of onchocerciasis appeared protective against STH infection (odds ratio 0.6, *P* < 0.05), with significantly lower STH prevalence among those LGAs at both timepoints (8.6% versus 12.4% in 2013 [*P* = 0.02] and 7.5% versus 10.8% in 2018 [*P* = 0.03]). However, when a difference-in-difference analysis was done, it showed that the changes in STH prevalence was not statistically significant between those from LGAs receiving annual ivermectin and those not (*P* = 0.53, Supplemental Figure 2).

There were no significant associations between STH infection and the number of years since the last community-wide LF MDA nor the cumulative years of LF MDA. The prevalence of STH among children living in LGAs that stopped LF MDA in 2012 versus 2009 was 10.5% versus 11.3% (*P* = 0.71) in 2013, and 9.8% versus 8.5% (*P* = 0.50) in 2018. However, the drop in STH infection seen from 2013 to 2018 in the LGAs that stopped LF MDA in 2009 was statistically significant (*P* = 0.03).

LGAs that had higher baseline prevalence of SCH received praziquantel annually instead of every other year. In 2013, those who would receive praziquantel every year had a prevalence of 16.0% compared with 12.1% in those who would receive it every other year; by 2018, the prevalence dropped to 13.0% versus 7.9%, respectively. The decreases in each group were statistically significant and although the prevalence decreased more in the group receiving MDA every other year, the interaction between treatment schedule and time was not statistically significant (*P* = 0.45).

### WASH data and associations with infection.

Questionnaires on student behavior and the school environment were collected in 2018. The WASH data were collected from 198/202 schools. Of these, 88 (44.4%) had latrines, 29 (14.7%) had facilities for handwashing, and 66 (33.3%) had a source of water. Of the schools with latrines, 67 (76.1%) had separate facilities for boys and girls. Most latrines were in poor condition, with 65.9% of latrines described as dirty and poorly maintained by data collectors. No schools had provisions for students to dry their hands after washing, and only one school provided soap.

**Figure 4.**
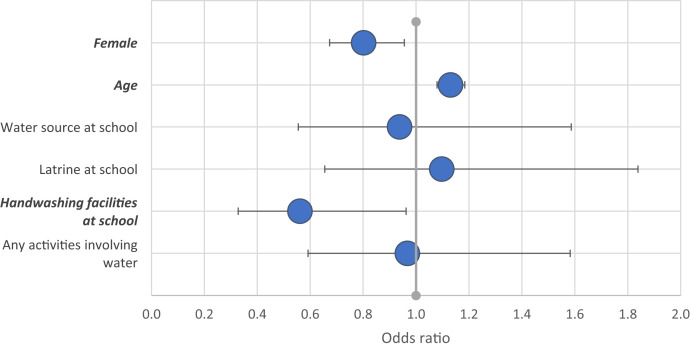
Odds ratio of a multivariable model exploring associations with schistosomiasis infection. Statistically significant variables are shown in bold. This figure appears in color at www.ajtmh.org.

**Figure 5.**
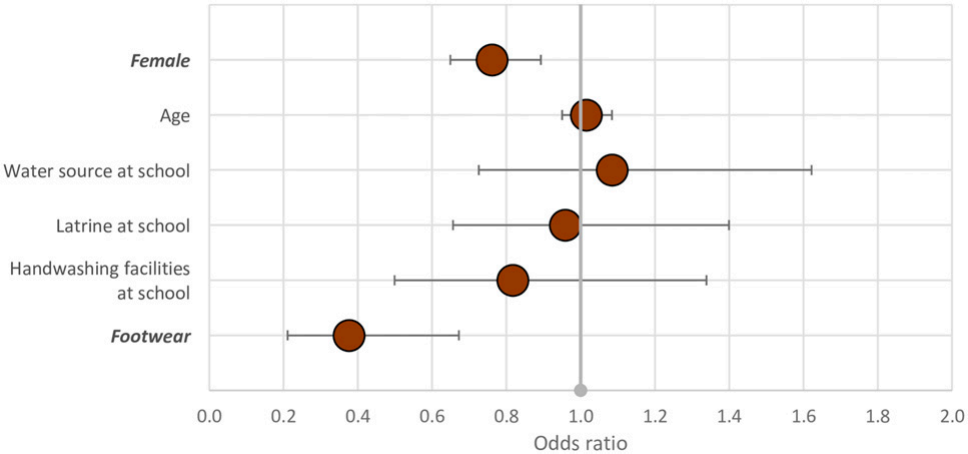
Possible associations with STH infection in 9,454 Nigerian children, 2018. Statistically significant variables are shown in bold italics. This figure appears in color at www.ajtmh.org.

We found that having SCH was less likely in girls and younger children aged 6–10 years, and there was an association with handwashing facilities (Figure 4). Girls were similarly less likely to have an STH infection, but there was no association with age or WASH variables. Wearing shoes appeared protective in this sample (Figure 5); 97% of children wore shoes, and 8% of those who did had hookworm, compared with 20% of those who did not (*P* = 0.03).

### Intensity of infection and dipstick sensitivity.

Most infections were of low intensity (Table 4). Heavy-intensity infections were uncommon in both samples (Table 5). Out of 30 LGAs surveyed, six were above the threshold of 1% prevalence of heavy infections for elimination of *S. hematobium* as a public health problem. Likewise, two of the 30 LGAs exceeded this 1% threshold for *S. mansoni* (Supplemental Table 1). Among children infected with *S. mansoni* (*N* = 962), heavy infections were found among 17.0% (95% CI: 9.7–28.2%) in 2013 and 7.5% (95% CI: 3.5–15.1%) in 2018. Across both studies, 1,430 children were positive for *S. hematobium*, either by dipstick or by urine filtration. Of children with *S. hematobium* identified by egg count (*N* = 239 or 2.5% [95% CI: 1.8–3.5%] of children in 2018), 21.3% (95% CI: 13.7–31.7%) had heavy infections. Heavy-intensity infections were found in < 1% of children with hookworm, and none were found in children with *A. lumbricoides* or *T. trichiura* in either timepoint in any LGA. When analysis was limited to children with hookworm, 0.5% (95% CI: 0.1–2.3%) of them had heavy infections in 2013, compared with 0.1% (95% CI: 0.0–0.8%) in 2018 (*P* = 0.3).

**Table 4:** Average parasite intensity among infected children from stool samples

State	Parasite	Mean* eggs per gram of stool (maximum)†	
		2013	2018
Nasarawa	*Ascaris lumbricoides*	782.4 (8,640)	459.7 (7,332)
	Hookworm	162.0 (3,720)	154.6 (6,240)
	*Schistosoma mansoni*	96.4 (1,440)	157.9 (1,200)
	*Trichuris trichiura*	88.0 (216)	32.0 (96)
Plateau	*A. lumbricoides*	202.0 (3,240)	172.7 (1,200)
	Hookworm	450.3 (24,480)	154.8 (2,796)
	*S. mansoni*	454.1 (11,520)	134.9 (2,316)
	*T. trichiura*	174.9 (576)	114.0 (192)

NB = thresholds for heavy-intensity infections were ≥ 400 eggs per gram (epg) of stool for *S. mansoni,* ≥ 50,000 for *A. lumbricoides*, ≥ 4,000 for hookworm, and ≥ 10,000 for *T. trichiura*.

* Means are calculated on positive children only.

† Average egg count per gram of stool was gathered through Kato-Katz technique and calculated by taking the average of two slide readings and multiplying it by 24.

**Table 5:** Frequency and prevalence of infection by intensity and organism in 2013 and 2018

Organism	Intensity of infection	2013 (%)	2018 (%)	Total
*Schistosoma hematobium*	Any	950 (8.4)	480 (4.2)	1,430
	Light	0 (0)	423 (3.7)	423
	Heavy	0 (0)	57 (0.5)	57
*Schistosoma mansoni*	Any	546 (4.8)	416 (3.7)	962
	Light	340 (3)	265 (2.3)	605
	Moderate	113 (1)	120 (1.1)	233
	Heavy	93 (0.8)	31 (0.3)	124
Hookworm	Any	1,093 (9.6)	823 (7.3)	1,916
	Light	1,074 (9.5)	816 (7.2)	1,890
	Moderate	13 (0.1)	6 (0.1)	19
	Heavy	6 (0.1)	1 (0)	7
*Ascaris lumbricoides*	Any	143 (1.3)	95 (0.8)	238
	Light	140 (1.2)	94 (0.8)	234
	Moderate	3 (0)	1 (0)	4
	Heavy	0 (0)	0 (0)	0
*Trichuris trichiura*	Any	10 (0.1)	12 (0.1)	22
	Light	10 (0.1)	12 (0.1)	22
	Moderate	0 (0)	0 (0)	0
	Heavy	0 (0)	0 (0)	0
Total				20,992

## DISCUSSION

We observed a significant reduction in the prevalence and intensity of SCH in school-aged children after 3–5 years of interventions in Nasarawa and Plateau states. There was also a modest reduction in the prevalence of STH infections over that time, with variable results across LGAs and schools. Prevalence of high-intensity infections was below 1% for all organisms and timepoints at the state level and in the majority of LGAs. The study draws on a large sample that allowed us to observe the effect of LGA-wide interventions on the same communities.

It is important to view the results of our 2013 and 2018 surveys in the context of other MDA programs. Praziquantel MDA was launched in two LGAs (Pankshin in Plateau state and Akwanga in Nasarawa state) in 1999 after heme dipstick mapping for *S. hematobium*.^7^ This scope of SCH control expanded modestly through additional dipstick mapping and praziquantel treatment until the E-Merck/WHO donation of praziquantel in 2008. This donation allowed higher frequency of treatment of more groups, not just those communities found through dipstick testing.^18^ Lymphatic filariasis MDA using ivermectin and albendazole was ubiquitous in all LGAs of the two-state area for the bulk of the 2000s, ending completely by 2013.^12^^,^^13^^,^^19^ Ivermectin was administered in many LGAs from the 1990s to 2017,^20^ and its ability to suppress STH infection is suggested here.^21^ Unfortunately, true baseline surveys for STH were not obtained to evaluate the impact of the LF program undoubtedly had on STH prevalence and intensity, but the end of LF treatment was a major impetus for performing the 2013 survey reported in this paper.^22^

As described elsewhere in Nigeria, we saw more SCH and STH infections in boys and older children.^23^^–^^28^ In contrast to other studies, comorbid infections were relatively uncommon, with only 2.1% of infected children having more than one STH.^29^^,^^30^ Hookworm was the most common STH and *T. trichiura* the least common in this sample, which is similar to other studies in Nigeria,^27^^,^^31^^–^^33^ and predictions from spatial models.^34^ *Ascaris lumbricoides*, the second-most frequent STH in these samples, has been the most common STH in other Nigerian contexts as well.^20^^,^^35^^,^^36^

Using two diagnostic methods for *S. hematobium* in 2018 affected our conclusions. Dipsticks more than doubled the number of positive children, with 241 children found negative through urine filtration but positive by dipstick test. When combined and using filtration-derived egg counts as the gold standard, dipsticks had a sensitivity of 93.7% and a specificity of 97.4%, but a positive predictive value of only 48.2%. Had they not been included, we would have seen a switch from SH to SM as the predominant organism, leading us to consider environmental changes that favored *Biomphalaria* snails over *Bulinus* species or differential effects of praziquantel on the two parasites. We also would have seen more dramatic drops in prevalence, had we only used one or the other test.

We sorted the LGAs into WHO’s treatment categories according the point estimates of prevalence. In 2013 (Figure 2), all 30 LGAs had SCH prevalence rates between 1% and 50%. By 2018, two LGAs had dropped into the lowest category (indicated in blue on the map). None had gone toward a “higher risk” category, indicated in orange or red on the map.

The picture for STH is more complicated. Given that there are more categories and organisms in play, it is not surprising that there is more shifting among them, especially as some LGAs’ prevalence increased between the two studies. In 2013, no LGAs were in the lowest risk category, and 16 were in the second lowest risk group (Figure 3). Five dropped to the lowest category and 10 in the second in 2018, and most LGAs were in the middle category of 10–20% prevalence.

Although the reduction in mean intensity of infection for most organisms was not statistically significant, we are encouraged that the range of intensities was much smaller in 2018. Comparing 2013–2018, the maximum egg count was 11,520 versus 2,316 for S. *mansoni*, 24,480 versus 6,240 for hookworm, and 576 versus 192 for *T. trichiura* in the two study years, respectively. The maximum count of *A. lumbricoides* showed less-impressive reductions, from 8,640 to 7,332, which is surprising given its sensitivity to mebendazole.^37^ Nonetheless, these results suggest an impact of the program on overall parasite intensity.

Most schools did not have adequate water and sanitation facilities, with 66% lacking latrines, 85% lacking facilities for handwashing, and 76% lacking a source of drinking water available to students. We do not know what sources of water were available to students outside of school. The majority of the population of these states did not have access to improved sanitation in 2013, but the proportions having access nearly doubled by 2018 to from 35.8% to 65.7% in Nasarawa and 18.8% to 35.6% in Plateau.^15^^,^^16^ Other studies have reported poor hygiene conditions in this area, with higher rates of infection than observed here.^38^ Provision of clean drinking water and sanitation infrastructure has been shown to reduce infection elsewhere in Nigeria,^31^^,^^39^ although the investment required for significant, sustained impacts on morbidity would be substantial.^31^^,^^39^ Shoes, which were worn by 97.1% of students in 2018, were protective here as they have been elsewhere, which is unsurprising given that hookworm was the most common STH.^40^ Our results show that MDA is helpful in reducing infection, but that it is insufficient on its own to completely eliminate these diseases as public health problems or to interrupt transmission. Investment in WASH infrastructure and behavior change will be needed to achieve these goals.

This study is limited by its focus on children attending school in an area with sub-optimal school enrollment. The prevalence of these ailments in the broader population could be quite different, especially given that low enrollment of children is associated with less adequate sanitation and higher rates of these infections.^41^ It is likely that, because MDA is based in schools, some children are systematically not reached, especially girls, who can be enrolled at lower rates than boys, such as in 2013 in Nasarawa state (69.3% versus 72.6% in 2013 and 67.2% versus 72.7% in 2018).^15^^,^^16^ However, this could be mitigated by the longstanding use of community-based treatment structures (i.e., volunteer community drug distributors) to reach children not in school, which occurs in some LGAs in these two states.^7^^,^^10^ We calculated prevalence using statistical procedures appropriate for cluster sampling, which widened our CIs. We were missing the original sample frames and thus could not incorporate weights. The study is subject to nonresponse bias because of insecurity that prevented revisiting some schools, as well as a lack of community- or individual-level treatment data, which could further explain the results.

These results could have implications for future MDA schedules per the WHO recommendations.^17^ As of 2018, 9 of the 30 LGAs could consider reducing the frequency of MDA based on the results of this study. However, since high-intensity schistosome infections remain, such decisions should be made with caution. The treatment algorithm is more complex for STH: based on 2018 results, nine LGAs could reduce the frequency of mebendazole treatment, whereas four could increase it. The other 17 could continue as before. Of note, three LGAs were conducting MDA for STH every year between 2013 and 2018, when only every other year was required. Given the odds or reinfection,^42^ particularly in areas with poor WASH infrastructure, decisions to lessen the intensity of MDA should not be made lightly.

## CONCLUSION

Schistosomiasis and STH in Nasarawa and Plateau states of central Nigeria remain public health problems in this context despite progress achieved through MDA over the past decade. Research into MDA coverage, targeting, and frequency, as well as efforts to improve water safety and sanitation, are needed to further control these diseases and thereby improve the health of these children and their communities.

## Supplemental Material


Supplemental materials
