# Supplementary material for: Impact of Three to Five Rounds of Mass Drug Administration on Schistosomiasis and Soil-Transmitted Helminths in School-Aged Children in North-Central Nigeria
Source: Am J Trop Med Hyg. 2022 May 16;107(1):132–42. doi: 10.4269/ajtmh.21-1207 (PMC9294711; doi:10.4269/ajtmh.21-1207)
Supplement: Supplementary file 1 [file tpmd211207.SD1.pdf]

Proposed supplemental materials

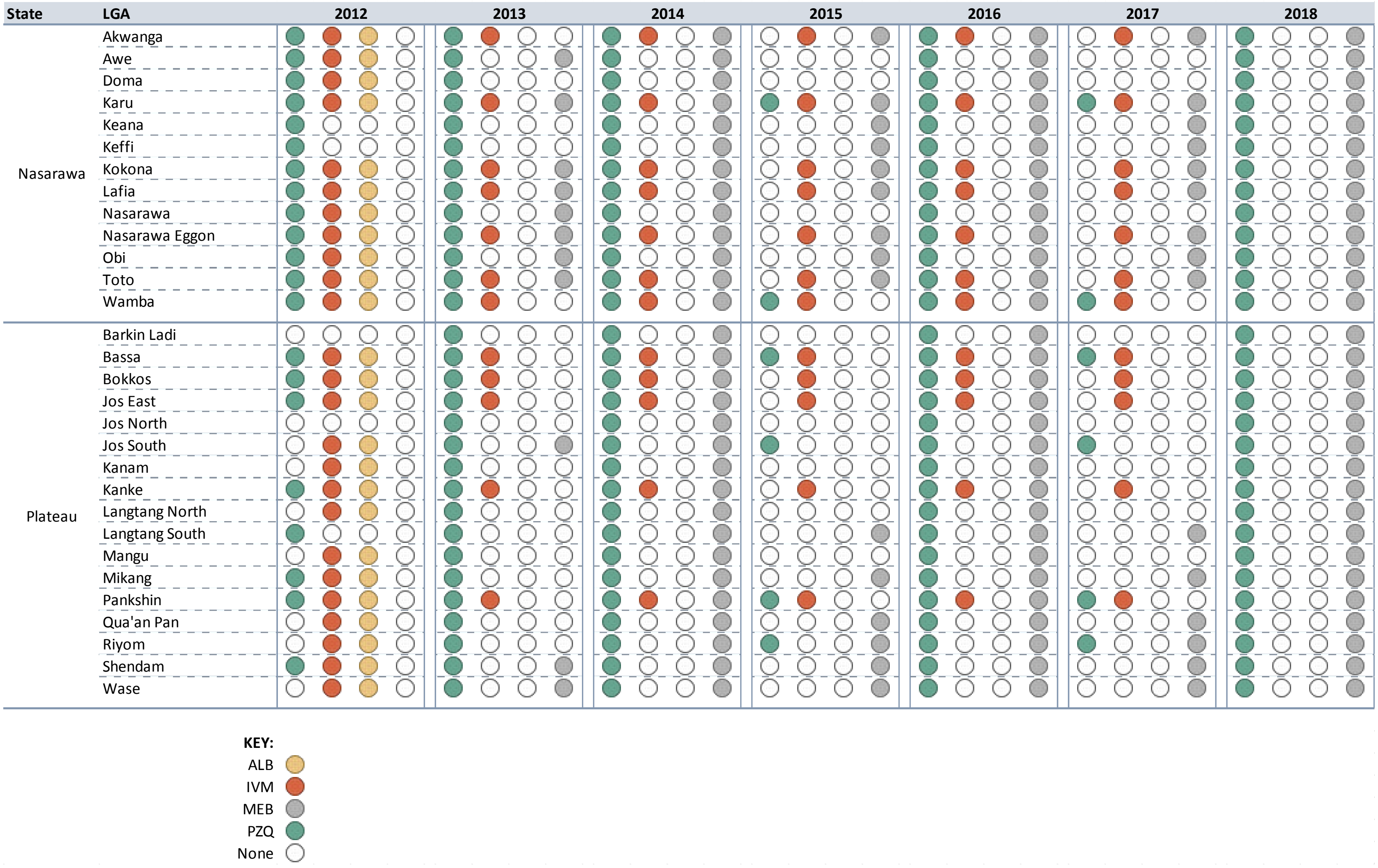

Supplemental Figure 1: Treatment history of 30 LGAs in Nasarawa and Plateau states, Nigeria, beginning the year before the survey (2012) and continuing through the year of the survey.

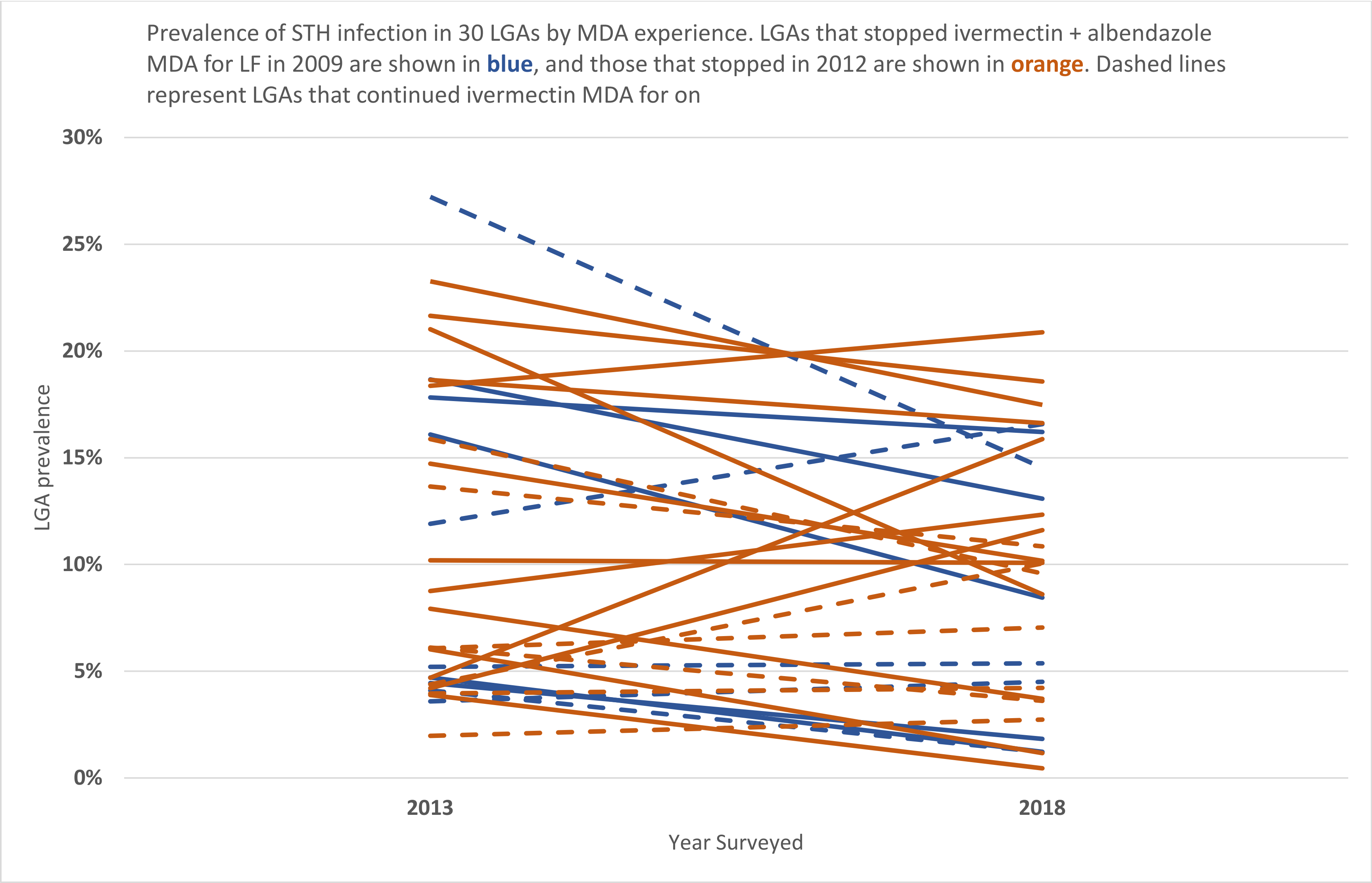

Supplemental Figure 2: Prevalence of STH infection in 30 LGAs, 2013 and 2018, categorized by exposure to MDA with ivermectin or ivermectin + albendazole.

Supplemental Table 1: Change in schistosomiasis prevalence by LGA from 2013 to 2018, both all infections and heavy infections only.

	S. haematobium prevalence		Prevalence of heavy-intensity *S. haematobium* infection		*S. mansoni* prevalence		Prevalence of heavy-intensity *S. mansoni* infection	
**State-LGA**	**2013**	**2018**	**2013**	**2018**	**2013**	**2018**	**2013**	**2018**
**Nasarawa**	**12.1%**	**7.1%**	**Not done**	**0.6%**	**3.5%**	**3.9%**	**0.1%**	**0.4%**
Akwanga	10.3%	4.5%		0.5%	3.3%	3.3%	0.0%	0.2%
Awe	25.8%	19.9%		3.3%	0.3%	0.0%	0.0%	0.0%
Doma	17.5%	14.4%		1.4%	0.0%	0.7%	0.0%	0.0%
Karu	16.1%	6.7%		0.0%	10.2%	19.5%	0.2%	3.6%
Keana	32.3%	0.9%		0.0%	0.3%	0.0%	0.0%	0.0%
Keffi	11.9%	6.3%		0.0%	2.9%	2.6%	0.0%	0.0%
Kokona	8.3%	6.1%		0.3%	2.8%	1.5%	0.0%	0.0%
Lafia	8.6%	7.8%		0.5%	3.6%	1.7%	0.0%	0.0%
Nasarawa	19.8%	21.1%		2.5%	4.8%	0.8%	0.3%	0.0%
Nasarawa Egon	2.7%	2.6%		0.0%	2.0%	2.3%	0.0%	0.0%
Obi	3.8%	4.5%		0.3%	0.7%	0.0%	0.2%	0.0%
Toto	7.6%	6.9%		1.1%	0.8%	0.0%	0.0%	0.0%
Wamba	1.0%	2.7%		0.0%	11.9%	7.3%	0.3%	0.3%
**Plateau**	**5.6%**	**3.6%**	**Not done**	**0.6%**	**6.2%**	**4.6%**	**1.3%**	**0.3%**
Barkin Ladi	0.5%	0.0%		0.0%	7.4%	0.4%	0.0%	0.0%
Bassa	1.5%	0.9%		0.0%	12.9%	16.4%	0.8%	1.2%
Bokkos	0.5%	0.9%		0.8%	8.0%	6.6%	1.6%	0.8%
Jos East	0.8%	2.0%		0.4%	5.4%	3.3%	0.3%	0.0%
Jos North	0.6%	1.8%		0.0%	5.8%	8.6%	0.9%	0.3%
Jos South	1.8%	0.4%		0.0%	10.3%	3.5%	1.3%	0.0%
Kanam	14.9%	12.9%		5.0%	0.2%	0.8%	0.2%	0.0%
Kanke	2.5%	1.7%		0.0%	1.3%	2.0%	0.2%	0.0%
Langtang North	20.7%	13.0%		2.6%	1.0%	1.3%	1.0%	0.0%
Langtang	1.9%	0.8%		0.0%	0.3%	0.5%	0.0%	0.0%

	***S. haematobium* prevalence**		**Prevalence of heavy-intensity *S. haematobium* infection**		***S. mansoni* prevalence**		**Prevalence of heavy-intensity *S. mansoni* infection**	
**State-LGA**	**2013**	**2018**	**2013**	**2018**	**2013**	**2018**	**2013**	**2018**
South								
Mangu	1.0%	0.9%		0.0%	7.6%	0.9%	1.7%	0.0%
Mikang	7.0%	3.5%		0.0%	5.8%	0.5%	0.0%	0.0%
Pankshin	0.5%	1.2%		0.0%	17.8%	8.2%	10.2%	0.0%
Qua'an Pan	3.3%	2.6%		0.0%	0.3%	1.5%	0.0%	0.0%
Riyom	0.0%	0.6%		0.0%	16.9%	9.4%	5.0%	0.6%
Shendam	16.4%	0.9%		0.0%	4.5%	3.2%	0.0%	0.6%
Wase	21.0%	13.2%		0.8%	1.2%	6.6%	0.9%	0.5%

.

Schisto School Form 1

River Blindness Nigeria 2017

User/Team

Observation Time

1. Form start time

2. School

2.1. *Choose a state, LGA, and school.
If the wrong options appear, you may have chosen incorrectly in the previous box.

Select One:
☐ Nasarawa ☐ Plateau

3. Students

3.1. Number of females enrolled in this school.

3.2. Number of males enrolled in this school.

3.3. Number of students sampled.
Must not be above 60.

4. Water in school

4.1. Is there a source of drinking water in the school?
Choose even if the source is not functioning - that will be asked later.

Select One:
☐ Yes ☐ No

ANSWER ONLY IF Question #4.1 is equal to "Yes"
4.2. What type(s) of water source(s)?

Select Multiple:
☐ Unprotected spring ☐ Protected spring
☐ Unprotected dug well ☐ Protected dug well
☐ Hand pump/tube well/borehole
☐ Surface water (river, dam, lake, stream, etc)
☐ Public piped water/tap/standpipe ☐ Rainwater collection
☐ Sachet/Pure water ☐ Other

ANSWER ONLY IF Question #4.2 includes "Other"
4.3. Specify "other" source of water.

ANSWER ONLY IF Question #4.1 is equal to "Yes"
4.4. Is the water source functional?

Select One:
☐ Yes ☐ No

ANSWER ONLY IF Question #4.4 is equal to "Yes"
4.5. Is the water always available for all school children and staff?

Select One:
☐ Yes ☐ No

5. Water near school

5.1. Are there sources of drinking water close to the school?

Select One:
☐ Yes ☐ No

ANSWER ONLY IF Question #5.1 is equal to "Yes"
5.2. What type(s) of water source(s)?

Select Multiple:
☐ Unprotected spring ☐ Protected spring
☐ Unprotected dug well ☐ Protected dug well
☐ Hand pump/tube well/borehole
☐ Surface water (river, dam, lake, stream, etc)
☐ Public piped water/tap/standpipe ☐ Rainwater collection
☐ Sachet/Pure water ☐ Other

ANSWER ONLY IF Question #5.2 includes "Other"
5.3. Specify "other" source of drinking water close to the school.

5.4. Are there accessible water bodies close to the school?

Select One:
☐ Yes ☐ No

6. Latrine

6.1. Is there a latrine in the school?

Select One:
☐ Yes ☐ No

ANSWER ONLY IF Question #6.1 is equal to "Yes"
6.2. INTERVIEWER: Do you observe evidence of latrine usage?
Data collector must personally check.

Select One:
☐ Yes ☐ No

ANSWER ONLY IF Question #6.1 is equal to "Yes"
6.3. What type of latrine is present?

Select One:
☐ Pit latrine without slab ☐ Pit latrine with slab
☐ Ventilated improved pit latrine (VIP) ☐ Flush or pour toilet
☐ Other

**ANSWER ONLY IF Question #6.3 includes "Other"**<br>6.4. Specify "other" latrine type.	
7. Latrine details	
7.1. What is the condition of the latrine?	**Select One:**<br>Poor (flies, odour, visible stool, etc.)<br>Fair (has roof/door but dirty floor)<br>Moderate (clean, absence of roof or door)<br>Good (clean, odourless, no flies, roof/door)<br>Excellent (clean, odourless, complete, water)
7.2. Is there a separate latrine for boys and girls?	**Select One:**<br>Yes No
7.3. Is there water or tissue for use after defecating?	**Select One:**<br>Always Sometimes Never Other
**ANSWER ONLY IF Question #7.3 is equal to "Other"**<br>7.4. Specify "other" kind of water or tissue for use after defecating.	
8. Handwashing	
8.1. Is there provision for hand washing after toilet use?	**Select One:**<br>Yes No
**ANSWER ONLY IF Question #8.1 is equal to "Yes"**<br>8.2. What type of hand washing facilities?	**Select One:**<br>No water Water only Water and soap<br>Water, soap, non-disposable napkin<br>Water, soap, disposable napkin Other
**ANSWER ONLY IF Question #8.2 is equal to "Other"**<br>8.3. Specify "other" type of hand washing facilities.	
8.4. Is there a hand washing facility near where food is prepared, sold, or consumed in school?	**Select One:**<br>Yes No
9. Health education	
9.1. Is health education taught in school?	**Select One:**<br>Yes No
9.2. Is there a health club in the school?	**Select One:**<br>Yes No
10. NTD program	
10.1. Has there been any deworming for the children in this school?	**Select One:**<br>Yes No
10.2. Is there a NTD treatment register in the school?	**Select One:**<br>Yes No
**ANSWER ONLY IF Question #10.2 is equal to "Yes"**<br>10.3. INTERVIEWER: Do you observe evidence of deworming in the treatment register?	**Select One:**<br>Yes No
**ANSWER ONLY IF Question #10.2 is equal to "Yes" AND Question #10.3 is equal to "Yes"**<br>10.4. Indicate the number of treatment rounds completed.	
11. Additional notes (optional).	
12. Form end time	

Schisto Student Form 1

River Blindness Nigeria 2017

User/Team	
Observation Time	
1. Form start time	
2. *****Choose a state, [illegible], and school.<br>*If the wrong options appear, you may have chosen incorrectly in the previous box.*	**Select One:**<br>☐ Nasarawa ☐ Plateau
3. Students	
3.1. *****Student UID<br>*Must be between 1 and 60.*	
3.2. Student UID second entry.<br>*Must be between 1 and 60.*	
3.3. Initials or name	
3.4. Gender	**Select One:**<br>☐ Male ☐ Female
3.5. Age (in years)<br>*Must be between 6 and 14 years.*	
3.6. How long have you lived in this community, in years?	
4. Urination	
4.1. When you are at school, where do you usually go to urinate?	**Select One:**<br>☐ In the school toilet ☐ Around the school compound<br>☐ Outside the school compound ☐ I wait/hold it ☐ Other
**ANSWER ONLY IF Question #4.1 is equal to "Other"**<br>4.2. Specify "other" options not mentioned above.	
5. Defecation	
5.1. When you are at school, where do you usually go to defecate?	**Select One:**<br>☐ In the school toilet ☐ Around the school compound<br>☐ Outside the school compound ☐ I wait/hold it ☐ Other
**ANSWER ONLY IF Question #5.1 is equal to "Other"**<br>5.2. Specify "other" options not mentioned above.	
6. Water	
6.1. If there are water bodies, which of the following activities do you perform?<br>*Ask the child what kinds of activities he/she engages in with water.*	**Select Multiple:**<br>☐ Bathing ☐ Washing ☐ Fishing<br>☐ Crossing the water ☐ Fetching water ☐ Playing<br>☐ Swimming ☐ Other
**ANSWER ONLY IF Question #6.1 includes "Other"**<br>6.2. Specify "other" options not mentioned above.	
7. Is the child putting on any footwear (sandals, shoes, slippers, etc.)?<br>*Observe if the child is wearing any form of shoes before responding.*	**Select One:**<br>☐ Yes ☐ No
8. Which samples were collected?<br>*Leave blank if no samples were collected from this child.*	**Select Multiple:**<br>☐ Stool ☐ Urine
9. Additional notes (optional).	
10. Form end time	
